# Glucose Uptake Stimulatory and PTP1B Inhibitory Activities of Pimarane Diterpenes from *Orthosiphon stamineus* Benth

**DOI:** 10.3390/biom9120859

**Published:** 2019-12-11

**Authors:** Phi Hung Nguyen, Huynh Nhu Tuan, Duc Thuan Hoang, Quoc Trung Vu, Minh Quan Pham, Manh Hung Tran, Dao Cuong To

**Affiliations:** 1 Institute of Natural Products Chemistry, Vietnam Academy of Science and Technology (VAST), 18 Hoang Quoc Viet, Cau Giay, Hanoi 122100, Vietnam; minhquanaries@gmail.com; 2 Graduate University of Science and Technology, VAST, 18 Hoang Quoc Viet, Cau Giay, Hanoi 122100, Vietnam; 3 Faculty of Pharmacy, Dong A University, 33 Xo Viet Nghe Tinh, Hai Chau District, Da Nang 550000, Vietnam; hnhutuandn@gmail.com; 4 Hanoi National University of Education, 136 Xuan Thuy, Cau Giay, Hanoi 123106, Vietnam; ducthuan75@gmail.com (D.T.H.); trungvq@hnue.edu.vn (Q.T.V.); 5 Biomedical Sciences Department, Institute for Research & Executive Education (VNUK), The University of Danang, 158A Le Loi, Hai Chau, Danang 551000, Vietnam; tmhung801018@gmail.com; 6 Faculty of Pharmacy, Phenikaa University, Yen Nghia, Ha Dong, Hanoi 12116, Vietnam; 7 Phenikaa Research and Technology Institute (PRATI), A&A Green Phoenix Group JSC, No.167 Hoang Ngan, Trung Hoa, Cau Giay, Hanoi 11313, Vietnam

**Keywords:** Lamiaceae, *Orthosiphon stamineus*, Pimarane diterpenes, Cat’s whisker, anti-diabetes

## Abstract

Seven pimarane diterpenes (**1**–**7**) were isolated from *Orthosiphon stamineus* Benth. by assay-guided isolation. All of the isolates possessed a 2-deoxy-2-((7-nitro-2,1,3-benzoxadiazol-4-yl)amino)-d-glucose uptake effect in 3T3-L1 adipocytes at concentrations of 5 and 10 μM. Most of them showed potent inhibition against protein tyrosine phosphatase 1B with IC_50_ values ranging from 0.33 to 9.84 μM. In the kinetic study, all inhibition types were exposed for the examined potencies, including mixed-competitive (**1**), non-competitives (**3** and **5**), competitive (**6**), and uncompetitive (**7**). The results suggested that *O. stamineus* and its pimarane diterpenes might exert the hypoglycemic effect via the insulin signaling pathway targeting inhibition of protein tyrosine phosphatase 1B (PTP1B) activity.

## 1. Introduction

Diabetes mellitus (DM) is a fast-growing metabolic disease affecting people globally. DM considered by hyperglycemia is referring to a high level of blood sugar. DM is mainly alienated into type 1 and type 2. Type 1 diabetes is insulin-dependent and mostly caused by the damage of pancreatic β cells that lead to the lack of insulin. Type 2 DM is non-insulin dependent diabetes mellitus. The lack of insulin is mainly an initial factor of type 2. The number of patients with type 2 DM has increased recently in developing countries because of the modern diet and genetic factors [1]. Currently, the treatments for type 2 DM depend on insulin injection and some hypoglycemic agents through oral administration. However, reports said that the long-term insulin injections to patients might cause several issues, such as pain and cost [2]. In addition, other therapeutics, such as oral hypoglycemic agents, resulted ineffectively. Nowadays, researchers all over the world attempt to find more safe and effective drugs in DM treatments. Among the DM therapies, protein tyrosine phosphatase 1B (PTP1B) is identified as an important point for type 2 DM treatment [3]. In the cell system, tyrosine phosphorylation and dephosphorization processes have been known as basic mechanisms of cell growth and differentiation. It is worthy to note that the stability of this progression is conserved by protein tyrosine phosphatases (PTPs) and protein tyrosine kinases (PTKs) [4]. PTP enzymes are specific, tightly regulated, and important modulators of cellular signal initiation and termination, which belong to the superfamily of receptor-like and non-transmembrane proteins. Among the PTPs members, PTP1B is a crucial member of the non-transmembrane PTPs family that negatively regulate insulin signal transduction [5]. PTP1B inhibition is proposed as an innovative target that specifically addresses insulin resistance sensitization. The inhibition of PTP1B also causes weight loss in obesity reduction, which is an important component of type 2 diabetic pathology [6]. Consequently, searching for PTP1B inhibitors, especially inhibitory agents from natural and medicinal plants, microorganisms, and animals, are the main prospects in both type 2 diabetes treatment and obesity [7,8]. There are many PTP1B inhibitors from medicinal plants. Up to now, around 56 families of genera from the natural source were found to inhibit PTP1B activity [9]. Among them, some plants and their secondary metabolites are derived from desert and steppe in the Middle East, such as *Artemisia judaica, Centaurium erythraea, Achillea santolina, Capparis spinosa, Moringa peregrina, Retama raetam, Terminalia chebula*, and *Ziziphus spina-christi* [10]. In addition, many chemical and pharmacological research works, and numerous natural compounds that could be used in anti-diabetic treatments through PTP1B inhibitory action have been identified in Chinese and Southeast Asian medicinal plants. In East Asia countries, such as China, Japan, Korea, Thailand, Vietnam, and Indonesia, the traditional medical treatment of diabetes often considers the integrated effects of different medicinal plants. A number of prescriptions directly impact different symptoms of diabetes. Most are from natural and medicinal plants [11], and a large number of experimental and clinical anti-diabetic activity research has been conducted [3,6,11]. According to recent reviews, approximately 500 natural secondary metabolites from the natural and medicinal plants could inhibit PTP1B activity via several pathways. They were classified in different chemical classes, such as fatty acids, phenolics, terpenoids, steroids, flavonoids, polysaccharides, and alkaloids [6,10,12].

*Orthosiphon stamineus* Benth. belongs to the *Orthosiphon* genus, which is one of the largest genus in the Lamiaceae family. This plant is widely distributed in Southeast Asia, Australia, and Africa [13], and is used for the treatment of many diseases in traditional medicines [14]. In Vietnam, this plant is named “Râu mèo”. The aerial parts of this plant were used in Vietnamese traditional medicine as an effective ingredient for the treatment of eruptive fever, hepatitis, urinary lithalsas, as well as influenza and jaundice [15]. In Indonesia, *O. stamineus* is known as a folk medicine usually to cure diabetes, and some inflammations, such as rheumatism, tonsillitis, and menstrual disorder. In addition, it was also used to reduce high blood pressure [16]. Previous studies on chemical constituents of *O. stamineus* showed the presence of essential oils [17], diterpenoids, triterpenoids, flavonoids, and phenolics [18,19]. Due to the plentiful chemical constituents, *O. stamineus* has been reported to have several pharmacological applications, such as anti-oxidant [18], anti-inflammatory [18,20], hepatoprotective [21,22,23], gastroprotective [21], anti-sebum [24], nephroprotective [18], antipyretic [25], and other diseases [26,27]. A few studies have shown the anti-diabetic effect of extract and fractions from *O. stamineus* [26,28]; however, there has been no investigation on the chemical constituents with glucose uptake stimulation and PTP1B inhibitory activity of *O. stamineus* to date. In this study, we report the isolation, structural elucidation, and the 2-NBDG uptake effects along with their PTP1B inhibitory activities of the pimarane diterpenes from *O. stamineus*.

## 2. Materials and Methods

### 2.1. General Experimental Procedures

Proton and carbon NMR were measured on a Varian Unity Inova 500 MHz spectrometer. The mass data were obtained from a Varian FT-MS spectrometer (Bruker Daltonics, Ettlingen Germany). Normal-phase and reverse-phase silica gels (F_254_, 40–63 mesh) were purchased from Merck, St. Louis, MO, USA). NP and RP TLC plates were from Merck. HPLC was carried out using a 1260 Agilent HPLC System: G1311C pump, G2260A auto-sampler, G1316A Thermo, and G1315D detector. Optima_Pak C18 column (10 × 250 mm, 10 and/or 5 µm particle sizes), RS Tech, Korea, and/or YMC-Pak ODS-AM (10 × 250 mm, 5 µm particle size) were used for purification.

### 2.2. Plant Material

*O. stamineus* material was obtained in 2017 in Hanoi, Vietnam. The plant sample, aerial parts, were identified by Dr. Quoc-Binh Nguyen of Vietnam National Museum of Nature, VAST. The specimen voucher for this plant was stored at INPC, VAST, Vietnam.

### 2.3. Isolation and Anti-Diabetes Assay Methods

The detailed extraction and isolation, NMR Spectroscopic data of the isolated compounds, cell culture and induction of 3T3-L1 adipocytes, cell viability assay, adipocyte-based measurement of 2-NBDG uptake, PTP1B inhibition assay, determination of the inhibition mode of active compounds, and statistical analysis are presented in the Supplementary Materials.

### 2.4. Statistical Analysis

Data are expressed as the mean ± standard deviation (SD). Statistical significance was assessed by the two-tailed unpaired Student’s *t*-test and P values less than 0.05 were considered statistically significant.

## 3. Results and Discussion

### 3.1. Structure Elucidation

The methanol extract of the aerial parts of *O. stamineus* was partitioned with chloroform (CHCl_3_), ethyl acetate (EtOAc), and butanol (BuOH) to obtain CHCl_3_, EtOAc, *n*-BuOH subfractions, and the H_2_O layer residue, respectively. Among these, the CHCl_3_ subfraction showed the strongest stimulation on 2-NBDG uptake assay in 3T3-L1 adipocytes (data not shown). Thus, this subfraction was chosen to isolate active principles.

**Figure 1:**
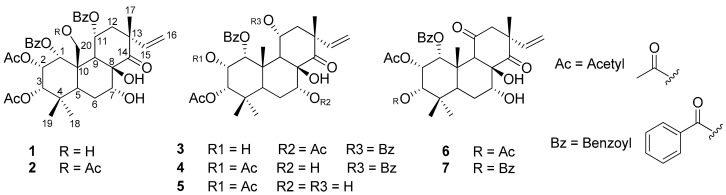
Structures of isolated compounds **1**–**7**.

Compound **1** was isolated as a colorless amorphous solid. The ^1^H-NMR spectrum of **1** displayed signals of three tertiary methyls (H-17, H-18, and H-19), three protons of vinyl group (H15, H16a, and H-16b), five oxygen-substituted methines (H-1, H-2, H-3, H-7, and H-11), one oxygen-substituted methylene (H-20), and two methylenes (H-6 and H-12), together with those of two acetyl and ten aromatic protons of benzoyl groups. Its ^13^C-NMR spectrum revealed that of a ketone signal (C-14), four carbonyls, seven oxygen-substituted, and three quaternary carbons (C-17, C-18, and C-19). In the COSY spectrum, the signals of ^1^H-^1^H cross-peak correlations between H-1/H-3 and H-2, H-6 and H-7, H-11 and H-12, as well as H-15 and H-16 were detected. In the HMBC spectrum, the proton H-1 at *δ*_H_ 5.60 (1H, d) displayed long-range correlations with carbon signals at *δ*_C_ 165.3 (C-7′), 68.2 (C-2), 76.9 (C-3), and 62.5 (C-20), suggesting the locations of benzoyl and oxygenated methylene groups at C-1 and C-20, respectively (Figure 1). Furthermore, a methine proton at *δ*_H_ 5.72 (1H, dd, H-2) was correlated with carbon signals at *δ*_C_70.6 (C-1), 76.9 (C-3), 38.0 (C-4), 49.8 (C-10), and 169.9 (2-OAc). The other methine proton at *δ*_H_ 4.97 (1H, d, H-3) was linked with carbons at *δ*_C_ 70.6 (C-1), 38.0 (C-4), 36.4 (C-5), 28.3 (C-18), 22.0 (C-19), and 170.7 (3-OAc) in the HMBC spectrum. These observations could suggest the location of two acetyl groups at C-2 and C-3. In addition, a methine proton at *δ*_H_ 5.88 (1H, m, H-11) exhibited long-range correlations with carbon signals at *δ*_C_ 77.6 (C-8), 49.8 (C-10), 48.4 (C-13), and 166.3 (C-7′′), indicating the second benzoyl group located at C-11. All the above data revealed that compound **1** was a pimarane-type diterpene [9,29,30,31] and was identical with siphonol A, except only for a lack of an acetyl moiety at C-7. The downfield chemical shift of methine proton H-7 (*δ*_H_ 5.51 ppm) in siphonol A [13] is quite different from the upfield chemical shift of methine proton H-7 (*δ*_H_ 4.32 ppm) in **1**. Based on the above evidence, compound **1** was thus identified as siphonol B [13].

The ^1^H- and ^13^C-NMR spectra of **2** were similar to those of **1** except for the presence of an acetyl group (*δ*_H_ 2.20 (3H, s, 20-OCOCH_3_), *δ*_C_ 171.2 (20-OCOCH_3_), and 21.2 (20-OCOCH_3_)) that is located at the C-20 position in **2** (Figure 1). This connection was confirmed by the HMBC correlations between three protons at *δ*_H_ 2.20 (3H, s) and two protons at *δ*_H_ 5.22 (1H, d, H-20a) and 4.14 (1H, d, H-20b) to the carbonyl carbon at *δ*_C_ 171.2. Therefore, **2** was identified as siphonol D [31].

Compounds **3**, **4**, and **5** were isolated as colorless amorphous solids. Their ^1^H-NMR spectrum displayed characteristic signals due to four tertiary methyls (H-17, H-18, H-19, and H-20), vinyl group (H-15 and H-16), and five oxygen-substituted methines (H-1, H-2, H-3, H-7, and H-11), together with those of acetyl and benzoyl groups (Figure 1). The ^13^C-NMR spectra of these compounds revealed the signals of a ketone (C-14), carbonyl carbons, six oxygenated carbons (C-1, C-2, C-3, C-7, C-8, and C-11), and three quaternary carbons (C-4, C-10, and C-13), which identified these compounds belonging to pimarane diterpene skeleton [13,29]. A detailed analysis of the ^1^H- and ^13^C-NMR spectra revealed that **3** possessed four carbonyl groups (1-OBz, 3-OAc, 7-OAc, and 11-OBz), two benzoyl units, one ketone (C-14), and six aliphatic oxygenated carbons. Compound **4** also possessed one ketone (C-14), six aliphatic oxygenated carbons, two benzoyl units, and four carbonyls carbons, but one of them was connected to C-2 (2-OAc) (Figure 1). Similar to **4**, compound **5** also possessed six aliphatic oxygenated carbons and one ketone (C-14) but showed only three carbonyl carbons (1-OBz, 2-OAc, and 3-OAc) (Figure 1). Comparing the ^1^H- and ^13^C-NMR data of these compounds to those published literature [13,30,31], the structures of **3**, **4**, and **5** were identified as orthosiphols B, F, and G, respectively.

Compound **6** was also isolated as a colorless amorphous solid. The ^1^H-NMR spectrum of **6** presented signals of four tertiary methyls (H-17, H-18, H-19, and H-20), four oxygen-substituted methines (H-1, H-2, H-3, and H-7), two acetyls, and two benzoyls (Figure 1). The ^13^C-NMR spectrum of **6** revealed the signals of two ketones (C-11 and C-14), three carbonyls (1-OBz, 2-OAc, and 3-OAc), five oxygenated carbons (C-1, C-2, C-3, C-7, and C-8), and three quaternary carbons (C-4, C-10, and C-13). Further analysis of these signals by the COSY and HMQC spectra led to the partial structures which were connected based on the long-range correlations observed in its HMBC spectrum. Thus, the structure of compound **6** was assigned to be orthosiphol I [30,31].

Compound **7** was also isolated as a colorless amorphous solid. The ^1^H- and ^13^C-NMR spectra of **7** were similar to those of **6** except for the replacement of an acetyl group in **6** by a benzoyl group located at C-3 in **7** (Figure 1). This connection was confirmed by the HMBC correlation between the proton H-3 (*δ*_H_ 5.31) and carbonyl carbon at *δ*_C_ 170.1. Therefore, compound **7** was identified as orthosiphol N [13].

### 3.2. 2-NBDG Assay Results

**Figure 2:**
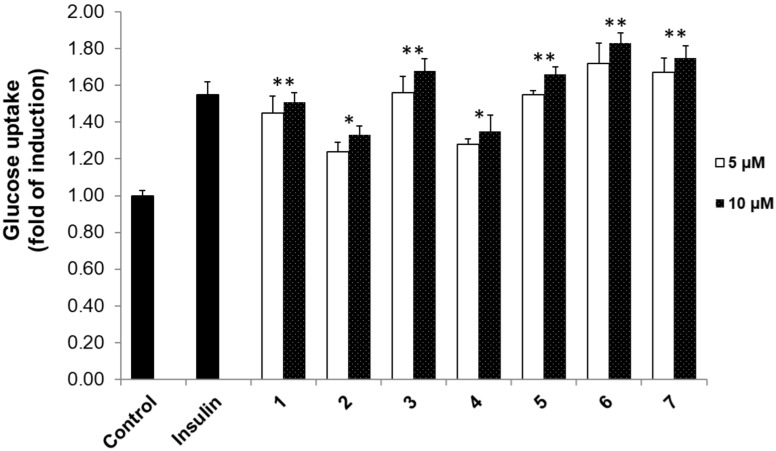
The action of pimarane diterpenes (**1**–**7**) on 2-NBDG uptake. Compounds concentrations were 5 and 10 µM; Insulin (100 nM); Control (DMSO). The glucose uptake effect was expressed as the mean ± standard deviation (SD) of three replicates. Statistical significance was accessed by Duncan’s multiple range tests (* *p* < 0.01; ** *p* < 0.05).

DM is a metabolic ailment that is categorized by glucose intolerance and deviations of lipid and protein metabolism processes. Previously, 2-NBDG was used as a potential agent to evaluate the action of compounds as insulin mimickers [32]. In our experiment, we examined the activity of the pimarane diterpenes **1**–**7** on 2-NBDG uptake in the 3T3-L1 adipocytes model. The results in Figure 2 show that these pimarane diterpenes exhibited stimulatory effects in dose-dependent manners. Among them, compounds **1**, **3**, **5**, **6**, and **7** theoretically stimulated glucose uptake by 1.45–1.51-, 1.56–1.68-, 1.55–1.66-, 1.72–1.83-, and 1.67–1.75-fold of inductions, respectively (Figure 2). Meanwhile, compounds **2** and **4** showed significant effects with 1.24–1.33- and 1.28–1.35-fold of inductions, respectively. We used insulin as a positive control, and it displayed a 1.55 ± 0.07-fold of induction at a concentration of 100 nM.

**Figure 3:**
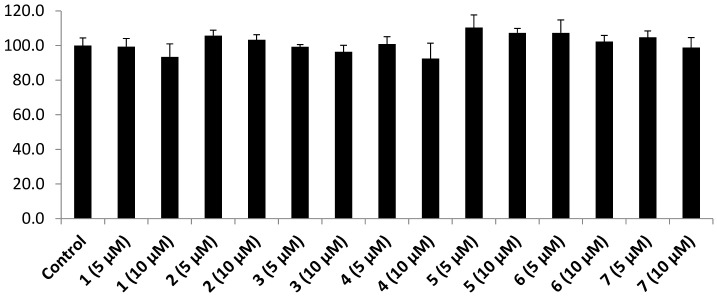
Cytotoxicity of pimarane diterpenes (**1**–**7**) on the viability of 3T3-L1 adipocyte cells.

The cytotoxicity of 3T3-L1 adipocyte cells affected by all isolated pimarane diterpenes was examined in the absence of 2-NBDG. The MTT assay in Figure 3 shows that no cytotoxicity was observed at both 5 and 10 μM of these compounds. These results might indicate that the stimulatory effects of **1**–**7** on 2-NBDG uptake were not pretentious by any cytotoxicity to the tested cells. Interestingly, compounds **6** (5 μM), and **7** (5 μM) showed stronger 2-NBDG uptake enhancements (1.72 ± 0.11- and 1.67 ± 0.08-fold of inductions) than insulin (1.55 ± 0.07) at 100 nM. Compounds **3** and **5** displayed a similar enhancing effect to the positive control with 1.56 ± 0.09- and 1.55 ± 0.02-fold of inductions at the same concentration of 5 μM, respectively. However, compounds **1**, **2**, and **4** (5 and 10 μM) showed less stimulatory effects than insulin with 1.51 ± 0.05, 1.33 ± 0.05, and 1.35 ± 0.09 μM, respectively.

### 3.3. Protein Tyrosine Phosphatase 1B Inhibition Results

PTP1B dephosphorylates IR and IRS in the insulin signaling pathway as a negative regulator. In the leptin signaling pathway, PTP1B presented the same manner of tyrosine Janus kinase 2. Thus, inhibition of PTP1B activity might improve the IR and IRS, leading to the enhancement of glucose uptake in adipocyte cells [32]. Hence, the diterpenes **1**–**7** were tested for their PTP1B inhibitory activity using the *p*-NPP substrate [12]. As a result, compounds **1**–**7** inhibited PTP1B activity in dose-dependent manners with IC_50_ values ranging from 0.33 ± 0.07 to 27.56 ± 2.99 μM (Table 1). Among the isolates, compound **6** showed the most potent inhibitory effect with an IC_50_ value of 0.33 ± 0.07 μM. Compound **7** also exhibited good inhibitory activity with an IC_50_ value of 1.60 ± 0.17 μM. Compound **5** displayed equivalent activity with an IC_50_ value of 3.82 ± 0.20 μM to ursolic acid (IC_50_ value of 3.42 ± 0.26 μM), the positive control. Compounds **1** and **3** inhibited PTP1B activity with IC_50_ values of 8.18 ± 0.41 and 9.84 ± 0.33 μM, respectively. Finally, compounds **2** and **4** displayed moderate activities with IC_50_ values of 24.75 ± 1.12 and 27.56 ± 2.99 μM, respectively.

### 3.4. Enzyme Kinetic Results

**Figure 4:**
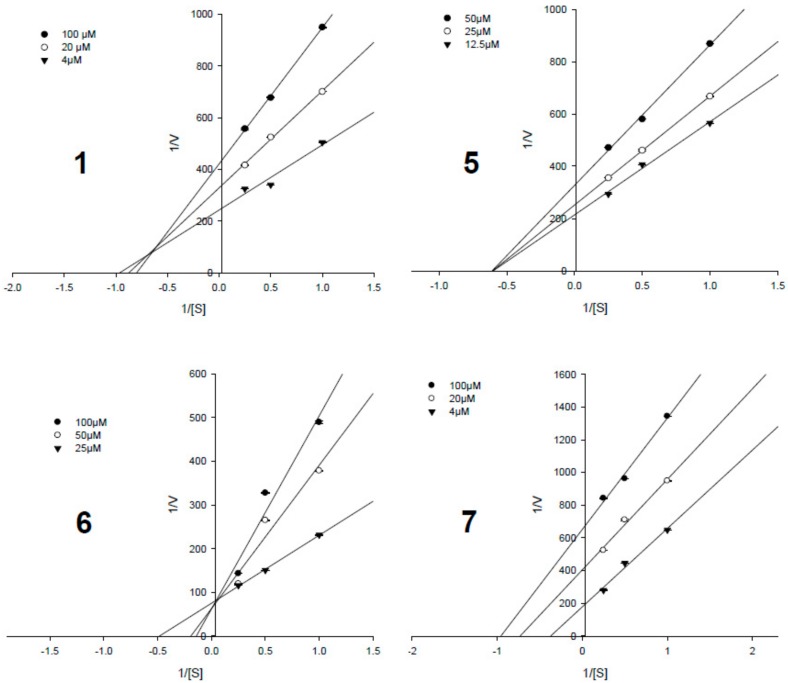
Lineweaver–Burk plots data of the active pimarane diterpenes (**1** and **5**–**7**).

**Figure 5:**
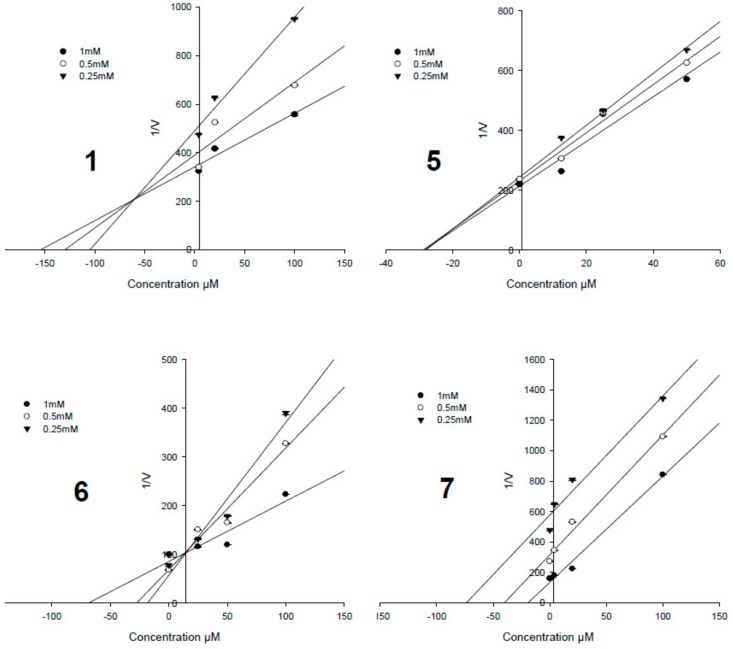
Dixon plots data of **1** and **5**–**7**.

To determine the enzyme inhibition types and inhibition constant (*K*_i_), the Lineweaver–Burk plot and Dixon plot [33,34] experiments were performed with the presence and absence of the active potencies (**1**, **3**, and **5**–**7**) with several concentrations of the substrate (*p*-NPP). The Sigma plot program was used to analyze both the double reciprocal Lineweaver–Burk plot and the Dixon plot by plotting 1/*v* as a function of the inhibitor (I). Figure 4 shows the results of the Lineweaver–Burk plot and Figure 5 shows the Dixon plot analyses for compounds **1**, **3**, **5–7**, respectively. Table 1 lists the *K*_i_ values for these active compounds. In this study, compound **6** (the most potency, the IC_50_ value of 0.33 ± 0.07 μM) showed a competitive inhibition mode due to its pattern of straight lines being in an intersection with the *y*-intercept (Figure 4 and Figure 5), indicating that **6** may directly bind to the active binding site of the PTP1B enzyme. Compound **7** (the second most potency, IC_50_ value of 1.60 ± 0.17 μM) showed uncompetitive inhibition, by increasing the substrate concentrations that resulted in a series of lines that did not intersect both on the *y*- and *x*-axis in the Lineweaver–Burk plot (Figure 4) and the *x*-axis in Dixon plots (Figure 5), but paralleled each other. Compounds **3** and **5** (IC_50_ values of 9.84 ± 0.33 and 3.82 ± 0.20 μM) possessed non-competitive inhibition based on the analyses of Lineweaver–Burk plot data and Dixon plots showing the lines intersected at a value of 1/(S) under zero on the *x*-axis (at 1/(intensity/min) = 0). This indicated that in allosteric inhibition, compounds **3** and **5** may bind to the enzyme-substrate complex or interact with a specific binding site distinct from the active site of the enzyme [35]. Only compound **1** (IC_50_ value of 8.18 ± 0.41 μM) inhibited PTP1B activity as a mixed-competitive inhibitor like ursolic acid due to the fact that the lines did not intersect at a value on the *y*-axis in the Lineweaver–Burk plot and the *x*-axis in Dixon plots (Figure 4 and Figure 5).

Based on the above results, it is worthy to note that those compounds, which stimulate effects on 2-NBDG uptake, also exhibit strong PTP1B inhibitory activity. Of our isolated pimarane diterpenes, compounds **6** and **7**, which have a ketone group at the C-11 position, displayed the strongest biological activities on both assays. Compound **6**, bearing a 3-*O*-acetyl moiety (at C-3) displayed stronger activity (IC_50_ = 0.33 μM) than compound **7** with a 3-*O*-benzoyl group (IC_50_ = 1.60 μM). In the same manner, compound **5**, with a hydroxyl group at the C-11 position, presented stronger inhibitory activity (IC_50_ = 3.82 μM) on PTP1B than compounds **3** (IC_50_ = 9.84 μM) and **4** (IC_50_ = 27.56 μM) with a benzoyl group at the same position, C-11. The above observation indicated that attachment of the ketone and/or hydroxyl groups (compounds **5**–**7**) at the C-11 position in the structure may have resulted in the enhancement of activity, and that substitution of the benzoyl group (compounds **1**–**4**) may be responsible for the decrease of activities of these pimarane diterpenes on both assays. Moreover, from a detailed investigation of the structure-activity relationship, we found that the attached position of the acetyl moiety on the structural skeleton of these pimarane diterpenes also played a significant role in the enhancement and/or decrease of the bioactivities. Indeed, compound **2**, bearing an acetyl moiety at the C-20 position, displayed less activity (IC_50_ = 24.75 μM) than compound **1** (IC_50_ = 8.18 μM) with C20-OH. In addition to this observation, compound **3**, having an acetyl moiety at C-7 and a hydroxyl group at C-2, showed stronger activity (IC_50_ = 9.84 μM) than compound **4** (IC_50_ = 27.56 μM), with opposition in the attachment of the acetyl and hydroxyl groups. Thus, it is suggested that the variation of the substituted positions of each functional group (acetyl, hydroxyl, and/or benzoyl) may lead to the variation of inhibitory effects on PTP1B activity and the stimulatory effects on 2-NBDG uptake of these diterpenes as well. These results also specified that the 2-NBDG uptake effects of the isolated diterpenes (**1**–**7**) on 3T3-L1 adipocytes maybe through their PTP1B inhibition. These results were in accordance with previous data that natural diterpene compounds were identified as potent PTP1B inhibitors. Accordingly, ent-pimara-8(14),15-diene-19-oic acid, 7-oxo-ent-pimara-8(14),15-diene-19-oic acid, 7β-hydroxy-ent-pimara-8(14),15-diene-19-oic acid, ent-pimara-8(14),15-diene-19-ol, 8α-hydroxy-ent-pimara-15-en-19-ol, and ent-kaur-16-en-19-oic-acid isolated from *Aralia continentalis* significantly inhibited PTP1B with very low IC_50_ values [36]. In the same manner with our data, these pimarane-type diterpenes showed mixed and noncompetitive inhibition against PTP1B with Ki values ranging from 3.2 to 12.8 μM. In particular, the compound 7-oxo-ent-pimara-8(14),15-diene-19-oic acid with an oxo group in the C-7 position might increase PTP1B inhibition due to the difference in its B ring component [36]. All these pimarane diterpenoids were chemically identified as pimara-8(14),15-diene in the skeleton. Interestingly, our diterpenoids are among the diterpene structure without diene linkage, and most of them have oxygenated functional groups connecting to C-1, C-2, C-3 or other carbon positions. However, both of the two sub-groups exhibited potential PTP1B, especially compound **6**, which bears one more 3-*O*-acetyl moiety at C-3, displayed the strongest PTP1B activity compared to the others. By discovering these above pimarane diterpenoids, this study provided an important opportunity to advance the understanding of the anti-diabetic drug discovery process.

## 4. Conclusions

In conclusion, this study described the isolation and structure elucidation of seven pimarane diterpenes (**1**–**7**) from *O. stamineus* as well as PTP1B inhibition of isolates. These compounds potentially inhibit PTP1B enzyme with IC_50_ values ranging from 0.33 to 9.84 μM. In addition, all pimarane diterpenes possess a 2-NBDG uptake effect in 3T3-L1 adipocytes cells. The kinetic study results showed that compound **1** exhibited mixed-competitive inhibition, compounds **3** and **5** showed non-competitive inhibition, competitive inhibition was shown for compound **6**, and uncompetitive inhibition for compound **7**. The results suggest that these active constituents from *O. stamineus* may be the potential natural products for the development of anti-hypoglycemic agents.

## Figures and Tables

**Table 1:** Protein tyrosine phosphatase 1B (PTP1B) inhibitory activity of pimarane diterpenes (**1**–**7**) isolated from *O. stamineus*.

Compounds	IC_50_, µM *^a^*	*K*_i_ Values, µM	Inhibition Type
**1**	8.18 ± 0.41	52.4 ± 0.9	Mixed-competitive
**2**	24.75 ± 1.12	- *^c^*	-
**3**	9.84 ± 0.33	75.6 ± 1.7	Non-competitive
**4**	27.56 ± 2.99	-	-
**5**	3.82 ± 0.20	23.9 ± 1.2	Non-competitive
**6**	0.33 ± 0.07	1.3 ± 0.6	Competitive
**7**	1.60 ± 0.17	5.5 ± 0.1	Uncompetitive
Ursolic acid *^b^*	3.42 ± 0.26	-	-

*^a^* IC_50_ values in µM, *^b^* Positive control. *^c^* Data not presented.

## Supplementary Materials

The following are available online at https://www.mdpi.com/2218-273X/9/12/859/s1, General experimental procedures; Extraction and isolation; NMR Spectroscopic data of isolated compounds (**1**–**7**); Cell culture and induction of 3T3-L1 adipocytes; Cell viability assay; Adipocyte-based measurement of 2-NBDG uptake; PTP1B Inhibition Assay; and Determination of the inhibition mode of active compounds.
