# Supplementary material for: Glucose Uptake Stimulatory and PTP1B Inhibitory Activities of Pimarane Diterpenes from Orthosiphon stamineus Benth
Source: Biomolecules. 2019 Dec 11;9(12):859. doi: 10.3390/biom9120859 (PMC7017366; doi:10.3390/biom9120859)
Supplement: Supplementary file 1 [file biomolecules-09-00859-s001.pdf]

Article

Glucose Uptake Stimulatory and PTP1B Inhibitory Activities of Pimarane Diterpenes from *Orthosiphon stamineus* Benth

Phi Hung Nguyen [1,2,†,*], Huynh Nhu Tuan [3,†],Duc Thuan Hoang [4], Quoc Trung Vu [4], Minh Quan Pham [1,2], Manh Hung Tran [5], and Dao Cuong To [6,7,*]

1. Institute of Natural Products Chemistry, Vietnam Academy of Science and Technology (VAST), 18 Hoang Quoc Viet, Cau Giay, Hanoi 122100, Vietnam; nguyenphihung1002@gmail.com (P.H.N.); minhquanaries@gmail.com (M.Q.P.)
2. Graduate University of Science and Technology, VAST, 18 Hoang Quoc Viet, Cau Giay, Hanoi 122100, Vietnam; nguyenphihung1002@gmail.com (P.H.N.); minhquanaries@gmail.com (M.Q.P.)
3. Faculty of Pharmacy, Dong A University, 33 Xo Viet Nghe Tinh, Hai Chau District, Da Nang 550000, Vietnam; hnhutuandn@gmail.com (H.N.T.)
4. Hanoi National University of Education, 136 Xuan Thuy, Cau Giay, Hanoi 123106, Vietnam; trungvq@hnue.edu.vn (Q.T.V); ducthuan75@gmail.com (D.T.H.)
5. Biomedical Sciences Department, Institute for Research & Executive Education (VNUK), The University of Danang, 158A Le Loi, Hai Chau, Danang 551000, Vietnam. tmhung801018@gmail.com (M.H.T.)
6. Faculty of Pharmacy, Phenikaa University, Yen Nghia, Ha Dong, Hanoi 12116, Vietnam; cuong.todao@phenikaa-uni.edu.vn (D.C.T.)
7. Phenikaa Research and Technology Institute (PRATI), A&A Green Phoenix Group JSC, No.167 Hoang Ngan, Trung Hoa, Cau Giay, Hanoi 11313, Vietnam; cuong.todao@phenikaa-uni.edu.vn (D.C.T.)

† These authors contributed equally to this research

* Correspondence: nguyenphihung1002@gmail.com (P.H.N); cuong.todao@phenikaa-uni.edu.vn (D.C.T)

Materials and Methods

General Experimental Procedures

^{1}H-NMR (500 MHz) and ^{13}C-NMR (125 MHz) were measured on a Varian Unity Inova 500 MHz spectrometer. ESI-MS was obtained from a Varian FT-MS spectrometer and MicroQ-TOF III (Bruker Daltonics, Ettlingen Germany). UV was performed in spectroscopic V-630 UV-VIS instrument. Column chromatography was carried out on silica gel (Si 60 F_{254}, 40-63 mesh, Merck, St. Louis, MO, USA). All solvents were redistilled before use. Pre-coated TLC plates (Si 60 F_{254}) were used for analytical purposes. Compounds were visualized under UV radiation (254, 365 nm) and by spraying plates with 10% H_2SO_4, followed by heating with a heat gun. The HPLC was carried out using a 1260 Agilent HPLC System with Quart Pump G1311C, auto-sampler G2260A, Column Thermo G1316A, and G1315D detector/DAD. The HPLC column of Optima_Pak C18 column was used (10 × 250 mm, 10 and 5 μm particle size), RS Tech, Korea, and/or YMC-Pak ODS-AM (10 × 250 mm, 5 μm particle size) for semi-preparative runs.

Plant Material

O. stamineus (aerial parts) were collected in January 2017 at NguHiep, Thanh Tri district, Hanoi, Vietnam. The plant sample was recognized by Binh Nguyen, PhD. (Vietnam National Museum of Nature). The specimen voucher of this plant is SH-164 and deposited at the Institute of Natural Products Chemistry (INPC), Vietnam.

Extraction and Isolation

The aerial parts (10 kg) were extracted with methanol (15 L × 3 times) at 40 °C using sonication for 5 h. After filtering and removing the solvent via rotary evaporator, the total extract was suspended in distilled water (1 L) and partitioned with $CHCl_3$ (1 L × 4 times), EtOAc (1 L × 4 times), and BuOH, successively. The resulting fractions were concentrated under reduced pressure in a rotary evaporator to give the $CHCl_3$, EtOAc, and BuOH fractions, and a H_2O residue, respectively. Following the 2-NBDG assay-guided fractionation, the $CHCl_3$ fraction was directly chromatographed on an open silica gel column (10 × 80 cm; 63–200 µm particle size, Merck) using a stepwise gradient of *n*-Hexane/acetone (from 20:1 to 2:1) to give five fractions (OSC-1 to OSC-5), according to their TLC profiles. Fraction 2 (OSC-2) was further fractionated by another open silica gel column (4.0 x 60 cm, 63–200µm particle size), eluted with a gradient solvent system of *n*-Hexane/EtOAc (from 15:1 to 5:1), and afforded five sub-fractions (OSC-2.1 to OSC-2.5). Sub-fraction OSC-2.2 was further purified by the Agilent 1260 HPLC system with an RS Tech Optima PakC_{18} column (10 × 250 mm, 10 µm particle size), and eluted with an isocratic solvent system of 70% ACN in H_2O + 0.1% formic acid (flow rate 2 mL/min) over 40 min, using UV detections at 205 and 254 nm, resulting in the isolation of compound **4** (12.9 mg; t_R = 29.6 min). Fraction 3 (OSC-3) was also fractionated by open silica gel column (3.0 × 60 cm, 63–200µm particle size), eluted with a gradient solvent system of *n*-Hexane/EtOAc (from 12:1 to 4:1), and afforded five sub-fractions (OSC-3.1 to OSC-3.5). Sub-fraction OSC-3.3 was further purified by the Agilent 1260 HPLC system with an RS Tech Optima PakC_{18} column (10 × 250 mm, 10 µm particle size), and eluted with an isocratic solvent system of 65% ACN in H_2O + 0.1% formic acid (flow rate 2 mL/min) over 30 min, using UV detections at 205 and 254 nm, resulting in the isolation of compounds **7** (27.8 mg; t_R = 19.6 min) and **3** (8.1 mg; t_R = 23.8 min), respectively. Fraction 4 (OSC-4) was also fractionated by the same open silica gel column (3.0 × 60 cm, 63–200µm particle size), eluted with a gradient solvent system of *n*-Hexane/EtOAc (from 10:1 to 2:1), and afforded five sub-fractions (OSC-4.1 to OSC-4.5). Purification of sub-fraction OSC-3.2 by the Agilent 1260 HPLC system using an RS Tech Optima PakC_{18} column (10 × 250 mm, 10 µm particle size) and eluted with an isocratic solvent system of 60% ACN in H_2O + 0.1% formic acid (flow rate 2 mL/min) over 40 min, using UV detections at 205 and 254 nm, resulted in the isolation of compound **1** (11.5 mg; t_R = 30.1 min) and compound **2** (5.8 mg; t_R = 35.6 min), respectively. Finally, compounds **5** (14.6 mg; t_R = 13.4 min) and **6** (23.3 mg; t_R = 18.1 min) were purified from sub-fraction OSC-4.4 by the Agilent 1260 HPLC system using the same HPLC described previously, and eluted with an isocratic solvent system of 58% ACN in H_2O + 0.1% formic acid (flow rate 2 mL/min) over 30 min, respectively.

*NMR Spectroscopic Data of Isolated Compounds (**1**–**7**)*

Siphonol B (**1**): colorless amorphous solid;^{1}H-NMR (500 MHz, Acetone-d_6) δ_H: 5.60 (1H, d, *J* = 2.0 Hz, H-1), 5.72 (1H, dd, *J* = 2.0, 4.0 Hz, H-2), 4.97 (1H, d, *J* = 4.0 Hz, H-3), 2.92 (1H, dd, *J* = 3.0, 13.5 Hz, H-5), 2.16 (1H, m, H-6a), 1.80 (1H, m, H-6b), 4.32 (1H, dd, *J* = 3.0, 6.0 Hz, H-7), 3.35 (1H, d, *J* = 5.5 Hz, H-9), 5.88 (1H, m, H-11), 2.77 (1H, dd, *J* = 4.0, 15.5 Hz, H-12a), 2.20 (1H, dd, *J* = 1.5, 15.5 Hz, H-12b), 5.76 (1H, dd, *J* = 10.5, 17.5 Hz, H-15), 4.88 (1H, dd, *J* = 0.5, 17.5 Hz, H-16a), 4.53 (1H, dd, *J* = 0.5, 10.5 Hz, H-16b), 1.23 (3H, s, H-17), 0.97 (3H, s, H-18), 1.18 (3H, s, H-19), 4.46 (1H, d, *J* = 13.0 Hz, H-20a), 4.14 (1H, d, *J* = 13.0 Hz, H-20b), 7.67 (2H, dd, *J* = 1.0, 8.5 Hz, H-2′/6′), 7.26 (2H, t, *J* = 8.5 Hz, H-3′/5′), 7.49 (1H, m, H-4′), 1.72 (3H, s, 2-OCOC$\underline{H}_3$), 1.50 (3H, s, 3-OCOC$\underline{H}_3$), 3.93 (1H, d, *J* = 3.0 Hz, 7-OH), 5.16 (1H, br s, 8-OH), 7.45 (2H, dd, *J* = 1.0, 8.0 Hz, H-2″/6″), 7.03 (2H, t, *J* = 8.0 Hz, H-3″/5″), 7.35 (1H, m, H-4″);^{13}C-NMR (125 MHz, Acetone-d_6) δ_C: 70.6 (C-1), 68.6 (C-2), 76.9 (C-3), 38.0 (C-4), 36.4 (C-5), 24.8 (C-6), 68.7 (C-7), 77.6 (C-8), 43.9 (C-9), 49.8 (C-10), 70.9 (C-11), 39.0 (C-12), 48.4 (C-13), 212.6 (C-14), 144.1 (C-15), 112.8 (C-16), 28.7 (C-17), 28.3 (C-18), 22.0 (C-19), 62.5 (C-20), 132.0 (C-1′), 130.7 (C2′/6′), 129.0 (C-3′/5′), 133.6 (C-4′), 165.3 (C-7′), 169.9 (2-O$\underline{C}$OCH$_3$), 20.7 (2-OCO$\underline{C}$H$_3$), 170.7 (3-O$\underline{C}$OCH$_3$), 20.6 (3-OCO$\underline{C}$H$_3$), 131.7 (C-1″), 130.4 (C-2″/6″), 128.6 (C-3″/5″), 132.9 (C-4″), 166.3 (C-7″).

Siphonol D (**2**): colorless amorphous solid;^{1}H-NMR (500 MHz, Acetone-d_6) δ_H: 5.67 (1H, d, *J* = 2.0 Hz, H-1), 5.44 (1H, dd, *J* = 2.0, 4.0 Hz, H-2), 4.98 (1H, d, *J* = 4.0 Hz, H-3), 2.97 (1H, dd, *J* = 3.0, 13.5 Hz, H-5), 2.83

(1H, m, H-6a), 1.83 (1H, m, H-6b), 4.43 (1H, dd, *J* = 4.0, 6.0 Hz, H-7), 3.35 (1H, d, *J* = 5.5 Hz, H-9), 6.05 (1H, m, H-11), 2.75 (1H, dd, *J* = 5.5, 15.0 Hz, H-12a), 2.04 (1H, dd, *J* = 1.0, 15.0 Hz, H-12b), 5.73 (1H, dd, *J* = 10.5, 17.5 Hz, H-15), 4.95 (1H, dd, *J* = 0.5, 17.5 Hz, H-16a), 4.58 (1H, dd, *J* = 0.5, 10.5 Hz, H-16b), 1.16 (3H, s, H-17), 0.99 (3H, s, H-18), 1.10 (3H, s, H-19), 5.22 (1H, d, *J* = 13.0 Hz, H-20a), 4.14 (1H, d, *J* = 13.0 Hz, H-20b), 7.76 (2H, dd, *J* = 1.5, 8.5 Hz, H-2′/6′), 7.37 (2H, t, *J* = 8.5 Hz, H-3′/5′), 7.57 (1H, m, H-4′), 1.74 (3H, s, 2-OCOC$\underline{H}_3$), 1.47 (3H, s, 3-OCOC$\underline{H}_3$), 4.43 (1H, br s, 7-OH), 5.10 (1H, br s, 8-OH), 7.53 (2H, dd, *J* = 1.0, 8.0 Hz, H-2′′/6′′), 7.11 (2H, t, *J* = 8.0 Hz, H-3′′/5′′), 7.42 (1H, m, H-4′′), 2.20 (3H, s, 20-OCOC$\underline{H}_3$);^{13}C-NMR (125 MHz, Acetone-d_6) δ_C: 70.9 (C-1), 68.4 (C-2), 76.6 (C-3), 37.9 (C-4), 36.3 (C-5), 24.6 (C-6), 69.9 (C-7), 78.2 (C-8), 42.4 (C-9), 48.1 (C-10), 68.2 (C-11), 40.0 (C-12), 48.4 (C-13), 211.3 (C-14), 143.8 (C-15), 113.4 (C-16), 27.7 (C-17), 28.5 (C-18), 22.6 (C-19), 63.9 (C-20), 132.0 (C-1′), 130.7 (C2′/6′), 129.0 (C-3′/5′), 133.6 (C-4′), 165.3 (C-7′), 170.0 (2-O$\underline{C}$OCH$_3$), 20.7 (2-OCO$\underline{C}$H$_3$), 170.7 (3-O$\underline{C}$OCH$_3$), 20.5 (3-OCO$\underline{C}$H$_3$), 131.7 (C-1′′), 130.4 (C-2′′/6′′), 128.6 (C-3′′/5′′), 132.9 (C-4′′), 166.3 (C-7′′), 171.2 (20-O$\underline{C}$OCH$_3$), 21.2 (20-OCO$\underline{C}$H$_3$).

Orthosiphol B (**3**): colorless amorphous solid;^{1}H-NMR (500 MHz, Acetone-d_6) δ_H: 4.92 (1H, d, *J* = 2.0 Hz, H-1), 4.43 (1H, dd, *J* = 2.0, 4.0 Hz, H-2), 4.95 (1H, d, *J* = 4.0 Hz, H-3), 2.50 (1H, dd, *J* = 2.5, 13.5 Hz, H-5), 2.10 (1H, m, H-6a), 1.96 (1H, m, H-6b), 5.47 (1H, dd, *J* = 2.0, 3.5 Hz, H-7), 3.30 (1H, d, *J* = 6.0 Hz, H-9), 5.88 (1H, m, H-11), 2.66 (1H, dd, *J* = 5.0, 15.5 Hz, H-12a), 1.95 (1H, dd, *J* = 1.0, 15.5 Hz, H-12b), 5.79 (1H, dd, *J* = 10.5, 17.5 Hz, H-15), 4.91 (1H, dd, *J* = 0.5, 17.5 Hz, H-16a), 4.77 (1H, dd, *J* = 0.5, 10.5 Hz, H-16b), 1.12 (3H, s, H-17), 0.88 (3H, s, H-18), 1.13 (3H, s, H-19), 1.57 (3H, s, H-20), 8.04 (2H, dd, *J* = 1.5, 8.0 Hz, H-2′/6′), 7.47 (2H, t, *J* = 8.0 Hz, H-3′/5′), 7.60 (1H, m, H-4′), 1.40 (3H, s, 3-OCOC$\underline{H}_3$), 2.19 (3H, s, 7-OCOC$\underline{H}_3$), 7.89 (2H, dd, *J* = 1.0, 8.0 Hz, H-2′′/6′′), 7.13 (2H, t, *J* = 8.0 Hz, H-3′′/5′′), 7.43 (1H, m, H-4′′);^{13}C-NMR (125 MHz, Acetone-d_6) δ_C: 79.1 (C-1), 66.2 (C-2), 79.2 (C-3), 38.0 (C-4), 37.4 (C-5), 22.0 (C-6), 71.3 (C-7), 76.0 (C-8), 42.4 (C-9), 48.1 (C-10), 69.8 (C-11), 40.7 (C-12), 48.5 (C-13), 207.6 (C-14), 143.5 (C-15), 113.7 (C-16), 26.5 (C-17), 28.2 (C-18), 23.0 (C-19), 17.0 (C-20), 132.0 (C-1′), 130.7 (C2′/6′), 129.0 (C-3′/5′), 133.6 (C-4′), 167.6 (C-7′), 170.7 (3-O$\underline{C}$OCH$_3$), 20.5 (3-OCO$\underline{C}$H$_3$), 169.9 (7-O$\underline{C}$OCH$_3$), 21.3 (7-OCO$\underline{C}$H$_3$), 131.7 (C-1′′), 130.4 (C-2′′/6′′), 128.6 (C-3′′/5′′), 132.9 (C-4′′), 166.3 (C-7′′).

Orthosiphol F (**4**): colorless amorphous solid;^{1}H-NMR (500 MHz, Acetone-d_6) δ_H: 5.21 (1H, d, *J* = 2.0 Hz, H-1), 5.52 (1H, dd, *J* = 2.0, 4.0 Hz, H-2), 4.97 (1H, d, *J* = 4.0 Hz, H-3), 2.83 (1H, dd, *J* = 2.0, 13.5 Hz, H-5), 2.11 (1H, m, H-6a), 1.81 (1H, m, H-6b), 4.39 (1H, dd, *J* = 3.5, 6.0 Hz, H-7), 3.20 (1H, d, *J* = 5.5 Hz, H-9), 5.68 (1H, m, H-11), 2.71 (1H, dd, *J* = 4.5, 15.5 Hz, H-12a), 2.02 (1H, dd, *J* = 2.0, 15.5 Hz, H-12b), 5.72 (1H, dd, *J* = 11.0, 17.5 Hz, H-15), 4.94 (1H, dd, *J* = 0.5, 17.5 Hz, H-16a), 4.66 (1H, dd, *J* = 0.5, 11.0 Hz, H-16b), 1.16 (3H, s, H-17), 0.96 (3H, s, H-18), 1.15 (3H, s, H-19), 1.53 (3H, s, H-20), 7.66 (2H, dd, *J* = 1.5, 8.0 Hz, H-2′/6′), 7.33 (2H, t, *J* = 8.0 Hz, H-3′/5′), 7.56 (1H, m, H-4′), 1.70 (3H, s, 2-OCOC$\underline{H}_3$), 1.47 (3H, s, 3-OCOC$\underline{H}_3$), 3.99 (1H, d, *J* = 4.5 Hz, 7-OH), 5.16 (1H, br s, 8-OH), 7.51 (2H, dd, *J* = 0.5, 8.0 Hz, H-2′′/6′′), 7.07 (2H, t, *J* = 8.0 Hz, H-3′′/5′′), 7.56 (1H, m, H-4′′);^{13}C-NMR (125 MHz, Acetone-d_6) δ_C: 73.1 (C-1), 67.5 (C-2), 76.9 (C-3), 38.2 (C-4), 36.2 (C-5), 24.7 (C-6), 68.4 (C-7), 78.1 (C-8), 42.4 (C-9), 49.8 (C-10), 70.1 (C-11), 40.3 (C-12), 48.3 (C-13), 211.4 (C-14), 143.9 (C-15), 113.2 (C-16), 27.4 (C-17), 28.2 (C-18), 22.8 (C-19), 17.5 (C-20), 131.8 (C-1′), 130.6 (C2′/6′), 128.9 (C-3′/5′), 133.4 (C-4′), 165.4 (C-7′), 170.2 (2-O$\underline{C}$OCH$_3$), 20.8 (2-OCO$\underline{C}$H$_3$), 170.7 (3-O$\underline{C}$OCH$_3$), 20.5 (3-OCO$\underline{C}$H$_3$), 130.6 (C-1′′), 130.3 (C-2′′/6′′), 128.5 (C-3′′/5′′), 132.9 (C-4′′), 166.3 (C-7′′).

Orthosiphol G (**5**): colorless amorphous solid;^{1}H-NMR (500 MHz, Acetone-d_6) δ_H: 5.64 (1H, d, *J* = 2.5 Hz, H-1), 5.50 (1H, dd, *J* = 2.5, 3.0 Hz, H-2), 4.97 (1H, d, *J* = 3.0 Hz, H-3), 2.75 (1H, dd, *J* = 2.0, 13.0 Hz, H-5), 2.01 (1H, m, H-6a), 1.72 (1H, m, H-6b), 4.23 (1H, m, H-7), 2.78 (1H, d, *J* = 8.5 Hz, H-9), 4.48 (1H, m, H-11), 2.26 (1H, dd, *J* = 5.0, 14.0 Hz, H-12a), 1.74 (1H, dd, *J* = 4.0, 14.0 Hz, H-12b), 5.92 (1H, dd, *J* = 11.0, 17.5 Hz, H-15), 4.90 (1H, dd, *J* = 1.0, 17.5 Hz, H-16a), 4.59 (1H, dd, *J* = 1.0, 11.0 Hz, H-16b), 1.15 (3H, s, H-17), 0.95 (3H, s, H-18), 1.15 (3H, s, H-19), 1.49 (3H, s, H-20), 8.10 (2H, dd, *J* = 1.5, 8.0 Hz, H-2′/6′), 7.47 (2H, t, *J* = 8.0 Hz, H-3′/5′), 7.58 (1H, m, H-4′), 1.89 (3H, s, 2-OCOC$\underline{H}_3$), 1.59 (3H, s, 3-OCOC$\underline{H}_3$);^{13}C-NMR (125 MHz, Acetone-d_6) δ_C: 75.2 (C-1), 67.9 (C-2), 77.2 (C-3), 38.2 (C-4), 36.1 (C-5), 24.8 (C-6), 68.8 (C-7), 78.5 (C-8), 45.0 (C-9), 44.5 (C-10), 64.7 (C-11), 45.0 (C-12), 49.3 (C-13), 212.3 (C-14), 143.3 (C-15), 113.8 (C-16), 26.7 (C-17), 28.4 (C-18), 23.0

(C-19), 16.8 (C-20), 133.0 (C-1′), 130.5 (C2′/6′), 129.0 (C-3′/5′), 133.1 (C-4′), 165.8 (C-7′), 170.5 (2-O$\underline{C}$OCH$_3$), 21.1 (2-OCO$\underline{C}$H$_3$), 170.7 (3-O$\underline{C}$OCH$_3$), 20.6 (3-OCO$\underline{C}$H$_3$).

Orthosiphol I (**6**): colorless amorphous solid;^{1}H-NMR (500 MHz, Acetone-d_6) δ_H: 6.35 (1H, d, J = 3.0 Hz, H-1), 5.57 (1H, dd, J = 3.0, 4.0 Hz, H-2), 4.50 (1H, d, J = 4.0 Hz, H-3), 2.67 (1H, dd, J = 2.0, 12.5 Hz, H-5), 2.04 (1H, m, H-6a), 1.77 (1H, m, H-6b), 4.23 (1H, m, H-7), 3.63 (1H, s, H-9), 2.65 (1H, d, J = 18.0 Hz, H-12a), 2.55 (1H, d, J = 18.0 Hz, H-12b), 5.41 (1H, dd, J = 10.5, 17.5 Hz, H-15), 4.71 (1H, dd, J = 1.0, 17.5 Hz, H-16a), 4.10 (1H, dd, J = 1.0, 10.5 Hz, H-16b), 1.06 (3H, s, H-17), 0.95 (3H, s, H-18), 1.14 (3H, s, H-19), 1.45 (3H, s, H-20), 8.10 (2H, dd, J = 1.5, 8.0 Hz, H-2′/6′), 7.52 (2H, t, J = 8.0 Hz, H-3′/5′), 7.56 (1H, m, H-4′), 1.86 (3H, s, 2-OCOC$\underline{H}_3$), 1.72 (3H, s, 3-OCOC$\underline{H}_3$);^{13}C-NMR (125 MHz, Acetone-d_6) δ_C: 74.4 (C-1), 67.0 (C-2), 76.9 (C-3), 38.0 (C-4), 35.5 (C-5), 24.8 (C-6), 68.7 (C-7), 78.6 (C-8), 52.4 (C-9), 43.8 (C-10), 207.4 (C-11), 48.1 (C-12), 45.0 (C-13), 209.6 (C-14), 140.8 (C-15), 115.8 (C-16), 25.5 (C-17), 28.3 (C-18), 22.7 (C-19), 17.0 (C-20), 131.7 (C-1′), 130.8 (C2′/6′), 129.2 (C-3′/5′), 133.8 (C-4′), 165.1 (C-7′), 170.1 (2-O$\underline{C}$OCH$_3$), 24.9 (2-OCO$\underline{C}$H$_3$), 170.8 (3-O$\underline{C}$OCH$_3$), 25.0 (3-OCO$\underline{C}$H$_3$).

Orthosiphol N (**7**):colorless amorphous solid;^{1}H-NMR (500 MHz, Acetone-d_6) δ_H: 6.43 (1H, d, J = 3.5 Hz, H-1), 5.74 (1H, dd, J = 3.5, 4.0 Hz, H-2), 5.31 (1H, d, J = 4.0 Hz, H-3), 2.86 (1H, dd, J = 3.0, 13.0 Hz, H-5), 2.10 (1H, m, H-6a), 1.82 (1H, m, H-6b), 4.44 (1H, m, H-7), 3.66 (1H, s, H-9), 2.65 (1H, d, J = 17.5 Hz, H-12a), 2.55 (1H, d, J = 17.5 Hz, H-12b), 5.42 (1H, dd, J = 10.5, 17.5 Hz, H-15), 4.79 (1H, d, J = 17.5 Hz, H-16a), 4.10 (1H, d, J = 10.5 Hz, H-16b), 1.23 (3H, s, H-17), 1.01 (3H, s, H-18), 1.11 (3H, s, H-19), 1.50 (3H, s, H-20), 8.04 (2H, dd, J = 1.5, 8.0 Hz, H-2′/6′), 7.47 (2H, t, J = 8.0 Hz, H-3′/5′), 7.60 (1H, m, H-4′), 1.86 (3H, s, 2-OCOC$\underline{H}_3$), 7.89 (2H, dd, J = 1.0, 8.0 Hz, H-2″/6″), 7.13 (2H, t, J = 8.0 Hz, H-3″/5″), 7.43 (1H, m, H-4″);^{13}C-NMR (125 MHz, Acetone-d_6) δ_C: 75.0 (C-1), 66.9 (C-2), 77.6 (C-3), 38.6 (C-4), 35.7 (C-5), 24.8 (C-6), 68.2(C-7), 78.5 (C-8), 52.2 (C-9), 44.3 (C-10), 208.0 (C-11), 48.9 (C-12), 50.0 (C-13), 208.9 (C-14), 140.7 (C-15), 116.5 (C-16), 25.8 (C-17), 28.5 (C-18), 23.0 (C-19), 17.5 (C-20), 131.7 (C-1′), 130.7 (C2′/6′), 129.0 (C-3′/5′), 133.6 (C-4′), 167.0 (C-7′), 170.1 (2-O$\underline{C}$OCH$_3$), 21.0 (2-OCO$\underline{C}$H$_3$), 130.8 (C-1″), 130.4 (C-2″/6″), 128.6 (C-3″/5″), 132.9 (C-4″), 170.1 (C-7″).

Cell Culture and Induction of 3T3-L1 Adipocytes

3T3-L1 cells were purchased from American Type Culture Collection (ATCC, Manassas, VA, USA) and grown in DMEM with 10% FCS. To induce differentiation, 3T3-L1 pre-adipocytes were cultured until confluence was reached (0 day), and the culture medium was replaced with a fresh induction medium containing 5 μg/mL insulin, 0.5 mM 3-isobutyl-1-methylxanthine (IBMX), and 1 μM dexamethasone (DEX) in DMEM with 10% FBS for 2 days. The medium was then replaced with a differentiation medium containing 5 μg/mL insulin and DMEM with 10% FBS every 2 days for up to 8 days until the cells were harvested.

Cell Viability Assay

The viability of cultured cells was assessed using an MTT assay. In brief, 3T3-L1 (1×10^4 cells/well) cells seeded in 96-well plates were treated with various concentrations of samples for 48 h. Then, MTT solution (1 mg/mL) was added to each well, and the cells were incubated at 37 °C for 1 h. Finally, DMSO was added to dissolve the formazan crystals. Absorbance was measured at 540 nm using a spectrophotometer (Immuno Mini NJ-2300, Japan).

Adipocyte-Based Measurement of 2-NBDG Uptake

3T3-L1 adipocytes grown in black 96-well plates were incubated with each sample for 24 h at 37 °C in a 5% CO_2 atmosphere. Subsequently, 250 μM 2-NBDG (dissolved in PBS with 1% BSA) was added to the cells and incubated for a further 30 min. After incubation, cells were washed two times with PBS to remove excess fluorescence in the wells. Then, fluorescence retained by the cells was measured using a PerkinElmer

Victor3 V 1420 Multilabel Plate Counter at an excitation and emission wavelength of 485 nm and 535 nm, respectively.

PTP1B Inhibition Assay

Protein tyrosine phosphatase 1B (human recombinant) was purchased from Biomol International LP, Plymouth Meeting, PA, USA, and the inhibitory activities of the tested samples were evaluated using the method as described in [31]. Briefly, 0.05–0.1 μg of PTP1B (BIOMOL International L.P., Plymouth Meeting, PA, USA) and 4 mM *p*-NPP in a buffer containing 1 mMdithiothreitol, 0.1 M NaCl, 1 mM EDTA, and 50 mM citrate (pH 6.0), with or without test compounds, were added as 100 μL of a final volume to each of the 96 wells. After the reaction mixture was incubated at 37 °C for 30 min, 10 M NaOH was added to quench the reaction. PTP1B enzyme activity was determined by the amount of produced *p*-nitrophenol at 405 nm. The nonenzymatic hydrolysis of the substrate was corrected by measuring the control, which did not contain the PTP1B enzyme.

Determination of the Inhibition Mode of Active Compounds

The Lineweaver–Burk plot and Dixon plot experiments were carried out in the presence and absence of the inhibitors with various concentrations of *p*-NPP as the substrate. The inhibition modes of the tested compounds were assessed on the basis of their inhibitory effects on the K_m (dissociation constant) and V_{max} (maximum reaction velocity) of the enzyme, which were determined by the Lineweaver–Burk plot experiment. The Lineweaver–Burk plot is the double reciprocal plot of the enzyme reaction velocity (V) versus the substrate (*p*-NPP) concentration ($1/V$ versus 1/(*p*-NPP)).

Statistical Analysis

Data are represented as means ± SD of at least three independent experiments performed in triplicated assays and determined by regression analysis. For statistical analysis of the data for single comparison, the significance between means was determined by the Student’s *t* test. Sigma Plot program version 11.0 was used for analysis of the kinetic data.
